# Characterization of HPMC and PEG 400 Mucoadhesive Film Loaded with Retinyl Palmitate and Ketorolac for Intravaginal Administration

**DOI:** 10.3390/ijms252312692

**Published:** 2024-11-26

**Authors:** Maryel E. Hernández-González, Claudia A. Rodríguez-González, Laura E. Valencia-Gómez, Juan F. Hernández-Paz, Florida Jiménez-Vega, Mauricio Salcedo, Imelda Olivas-Armendáriz

**Affiliations:** 1 Departamento de Física y Matemáticas, Instituto de Ingeniería y Tecnología, Universidad Autónoma de Ciudad Juárez, Ciudad Juárez 32320, Mexico; maryel.hernandez@uacj.mx (M.E.H.-G.); claudia.rodriguez@uacj.mx (C.A.R.-G.); laura.valencia@uacj.mx (L.E.V.-G.); juan.hernandez.paz@uacj.mx (J.F.H.-P.); 2 Departamento de Ciencias Químico Biológicas, Instituto de Ciencias Biomédicas, Universidad Autónoma de Ciudad Juárez, Ciudad Juárez 32315, Mexico; fjimenez@uacj.mx; 3 Unidad de Investigación Biomédica Oncológica Genómica, Hospital Gineco Pediatría 3-A, Instituto Mexicano del Seguro Social, Ciudad de México 07790, Mexico; masava89@gmail.com

**Keywords:** HPMC, PEG 400, film, retinyl palmitate, intravaginal administration

## Abstract

Intravaginal drug administration offers several advantages over other routes, primarily bypassing the initial stages of metabolism. Additionally, this route has demonstrated both local and systemic effects. Mucoadhesive polymeric systems can be utilized to prevent dose loss due to the mucous barriers and the formation of wet cavities. This study employed various techniques to evaluate the performance and characteristics of a mucoadhesive film composed of HPMC-PEG 400 containing retinyl palmitate and ketorolac molecules. Scanning Electron Microscopy (SEM) was employed to analyze the porous structure of the film. Thermogravimetric Analysis (TGA) was conducted at different temperatures to assess thermal stability. Fourier Transform Infrared Spectroscopy (FTIR) was used to analyze the functional groups and intermolecular interactions between the film and the drug. Swelling and weight loss tests indicated that the film disintegrated within 3–4 days. UV-VIS spectroscopy was used for drug release evaluation based on the Higuchi equation. Additionally, the surface wetting properties were assessed through contact angle measurements. The system’s biocompatibility was confirmed using the MTT assay. Finally, adhesion and glide tests demonstrated the film’s interaction with porcine uterine tissue. This study shows that the HPMC-PEG 400 film containing retinyl palmitate molecules interacts effectively with tissue and could be considered a novel tool for treating damaged epithelial tissues.

## 1. Introduction

Ailments affecting the genital area in women are common due to various factors, including physical trauma from blows to the groin area, rape, childbirth, foreign objects, everyday incidents such as trauma during consensual sexual activity, wearing uncomfortable clothing, tampon use, and other causes like sexually transmitted infections (STIs) and recurrent urinary tract infections. Even menopause can disrupt the delicate balance needed for vaginal health [1,2,3,4,5]. According to Dolan et al. [2], most local trauma to the lower genital tract results from consensual sexual intercourse, particularly the tearing of the posterior fornix; these injuries typically occur in women aged 16 to 25 and those over 45. Approaches to addressing STIs vary among research groups. For instance, Notario-Perez’s group focuses on prevention [6], while Mishra’s group targets symptom management [7]. Treatment procedures depend on the type of infection, with different drugs loaded into carrier systems for conditions such as candidiasis [8], HIV [9], HPV [10], or microbial infections [11]. Many of these conditions and traumas result in lacerations to the vaginal tissue, necessitating active molecules that can promote cell growth, particularly in epithelial cells, such as retinol [12]. Retinol (Vitamin A) is present in the bloodstream through the ingestion of carotenoids [13] and is stored for use when needed. It is crucial in expressing several proteins and in overall lung health, vision, and fetal development [14].

Due to its anti-aging effects when applied to the skin and its role as a nutrient for vision and lung health, many research groups and companies are exploring retinol for epithelial tissue regeneration [15]. Various conditions, such as low intake, inflammation, prenatal periods, and infections, can lead to decreased levels of retinol in the blood, resulting in imbalances in epithelial tissue and an increased risk of infections [16].

It has been demonstrated that using retinol for cervicovaginal treatment can reverse metaplasia and promote cell proliferation [17]. A nonsteroidal anti-inflammatory drug (NSAID) can be administered to address inflammation and pain. One of the most versatile options available is ketorolac. It is an analgesic with anti-inflammatory effect that produces minimal tissue irritation. These effects have been observed in the vaginal area following illness, surgical procedures, or injury. This drug is rapidly absorbed when administered orally and intranasally [18]. Its clinical utility has been demonstrated in pain states such as osteoarthritis, rheumatoid arthritis, and metastatic bone cancer, and it is sometimes used as an alternative to opioids. Further, ketorolac has also been used for severe pain following abdominal, gynecological, orthopedic, and dental surgeries. Various studies have estimated its analgesic potency to be between 180 and 800 times more than aspirin, and to be as effective as morphine [19,20,21]. A loading dose of 30–60 mg is recommended, followed by 15–30 mg every 6 h, with its use limited to 5 days [22]. Ketorolac is classified as a nonselective cyclooxygenase (COX) inhibitor and can reduce pain perception within 48 h when applied intravaginally [23].

The earliest recorded use of vaginal administration for therapeutic purposes dates to ancient Egypt, where remedies were applied to address women’s health issues and aid in recovery from pelvic trauma following accidents or childbirth [24]. Since then, many treatments have been administered via this route due to its rich blood supply, which promotes both local and systemic responses. This method offers numerous benefits, such as allowing lower doses, less frequent administration, and steady drug levels, while also avoiding the first-pass effect, which means it does not impact the gastrointestinal system [25].

Various forms of intravaginal drug delivery have been introduced, including ovules, sponges, tablets, creams, gels, vaginal rings, and films [25]. Recently, an additional feature has been incorporated into these systems: mucoadhesiveness. This characteristic helps reduce leaks and the loss of active molecules by adhering to the mucosal surface [26]. Consequently, many research groups are developing mucoadhesive systems for intravaginal delivery to treat or prevent various conditions, especially STIs [27]. For a mucoadhesive system to effectively deliver and release active molecules, it must be made from biocompatible, biodegradable, and flexible materials, which is why polymers are often used [28]. Polymers are versatile macromolecules that bind to DNA and polypeptides through electrostatic interactions, enhancing the delivery of therapeutic molecules. They also exhibit excellent compatibility with living systems [29].

Hydroxypropyl methylcellulose (HPMC) is one of the most widely used drug-delivery polymers. Its hydrophilic properties allow it to swell upon contact with human fluids, leading to controlled release as the matrix degrades [30,31]. HPMC is often used in mucoadhesive polymeric films for controlled drug release in mucosal areas, such as the vagina [27]. Typically, HPMC is combined with a plasticizer-like polyethylene glycol (PEG 400). This low-molecular-weight, low-toxicity, and highly hydrophilic chain optimizes the film’s mechanical properties, providing flexibility to counteract the polymer’s stiff and brittle behavior at temperatures below the glass transition temperature. Plasticizers enhance workability, lubricate, reduce melt viscosity, and improve the polymer’s efficiency [32].

This investigation aimed to develop a mucoadhesive polymeric film using HPMC and PEG 400, loaded with retinyl palmitate and ketorolac for intravaginal administration.

## 2. Results

### 2.1. Colors and Textures of Films

**Figure 1:**
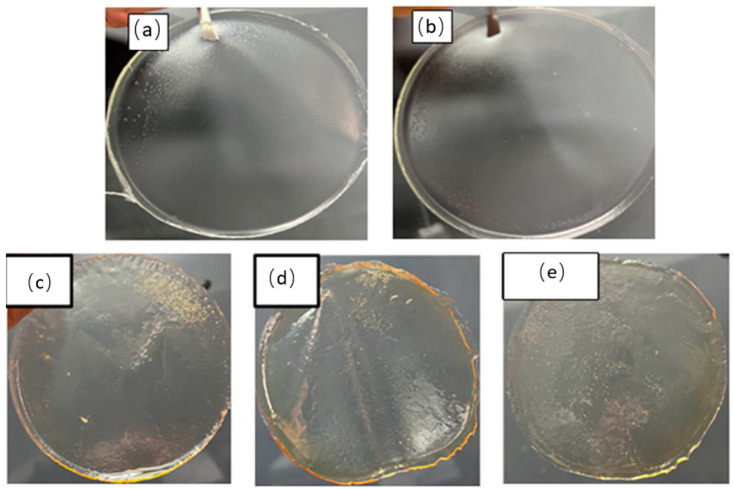
Polymeric films with different concentrations of HPMC and retinyl palmitate. (**a**) 15 wt. % of HPMC, 85 wt. % of PEG 400, and without retinyl palmitate (film A), (**b**) 21 wt. % of HPMC, 79 wt. % of PEG 400, and without retinyl palmitate (film C), (**c**) 20.94 wt. % of HPMC, 79 wt. % of PEG 400, and 0.67 wt. % of retinyl palmitate (film D), (**d**) 20.90 wt. % of HPMC, 78.38 wt. % of PEG 400, and 0.83 wt. % of retinyl palmitate (film E), and (**e**) 20.88 wt. % of HPMC, 78.10 wt. % of PEG 400, and 1 wt. % of retinyl palmitate (film F).

First, polymeric films were obtained, exhibiting various colors and textures depending on HPMC and retinyl palmitate concentrations. Visual inspection revealed that increasing the amount of HPMC resulted in the formation of larger bubbles when retinyl palmitate was added (Figure 1). The films also displayed increased moisture content and yellowness, with larger bubbles or pores becoming visible, which can enhance the adhesion of the film to the vaginal tissue.

### 2.2. Characterization Through Scanning Electron Microscopy

**Figure 2:**
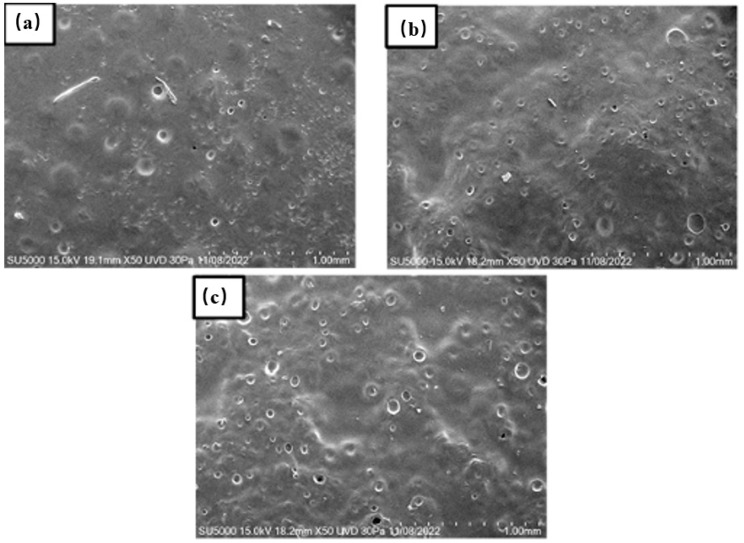
FE-SEM of polymeric films with (**a**) 15 wt. % of HPMC and 85 wt% of PEG 400 (film A), (**b**) 21 wt. % of HPMC and 79 wt. % of PEG 400 (film C), and (**c**) 20.94 wt. % of HPMC, 78.38 wt. % of PEG 400, and 0.67 wt. % of retinyl palmitate (film D).

The films demonstrated a porous topography with varying dimensions, from micro to macro pores, both closed and open, as observed using the FESEM technique. Our findings align with Sampath et al., indicating that the variety of pores can influence the material’s success in application [33]. In contrast to previous reports [27], the pores in our films were smaller than 100 µm. The pore size and shape varied with HPMC concentration (Figure 2a,b). For films containing 15 wt. % of HPMC, pore sizes ranged from 5 µm to 11 µm, with most pores being closed. In films with 21 and 20.94 wt. % of HPMC, most pores were open, ranging from 1 to 42 µm. Thus, it is possible to obtain films with both micro and macro pores. Additionally, the second film had a greater number of pores on its surface.

**Figure 3:**
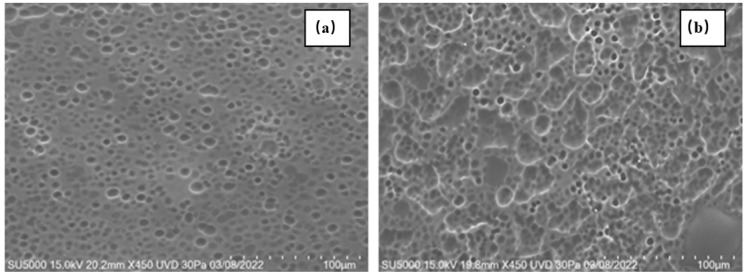
FE-SEM of polymeric films with (**a**) 20.90 wt. % of HPMC, 78.27 wt. % of PEG 400, and 0.83 wt. % of retinyl palmitate (film E) and (**b**) 20.88 wt. % of HPMC, 78.10 wt. % of PEG 400, and 1 wt. % of retinyl palmitate (film F).

When retinyl palmitate was added to the films, the surface morphology changed. Increasing the concentration of retinyl palmitate resulted in larger pores and topographical changes, making the system lumpier. This change in topography could improve the system’s adhesion to the vaginal epithelium. In Figure 2c, the pores had an average diameter of 24 µm, while the lumps ranged from 100 to 200 µm. For 0.83 wt. % of retinyl palmitate (Figure 3a), the pores were approximately 46 µm, and the lumps measured 445 µm. For 1 wt. % of retinyl palmitate (Figure 3b), the pores measured 70 µm, and the lumps ranged from 1500 to 3000 µm. These data indicate that increasing the concentration of retinyl palmitate alters the pore size and topography, resulting in a lumpier system.

The thickness of the HPMC and PEG 400 film was 155.6 ± 3.61 µm, whereas the thickness of the film containing HPMC, PEG 400, and retinyl palmitate measured 275.6 ± 16.98 µm. These thicknesses fall within the range reported in other studies, typically ranging from 50 to 200 µm. A lower thickness may compromise drug loading and structural integrity, while a greater thickness could affect the film’s flexibility and comfort [8].

### 2.3. TGA Analysis

**Figure 4:**
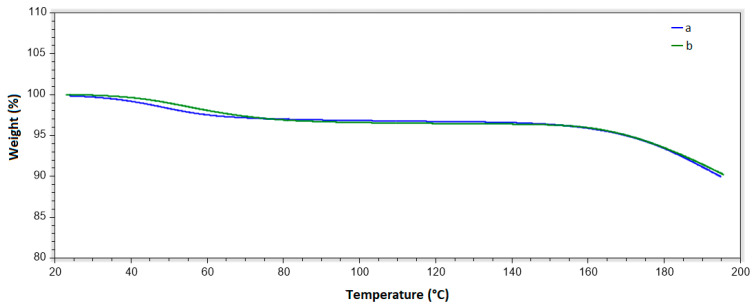
TGA spectrum. (a) 21 wt. % of HPMC and 79 wt. % of PEG 400 films without retinyl palmitate (film C), (b) 20.90 wt. % of HPMC and 78.27 wt. % of PEG 400 film with 0.83 wt. % of retinyl palmitate (film E).

Figure 4 presents the thermogravimetric analysis of films with and without retinyl palmitate, heated under controlled conditions (10 °C/min) from room temperature to 200 °C. Both materials exhibit similar behavior, showing at least two distinct thermal events. The DTG curves reveal a drying event (with a weight loss of about 2.5%) that begins at around 30 °C and ends at approximately 80 °C, likely due to the minimum presence of water and dichloromethane residues, the solvent used, which has a boiling point of 39.6 °C [34]. The second event, which starts at around 130 °C, corresponds to the decomposition of the film and continues until the end of the analysis. These findings are consistent with those reported in the literature, which attribute the weight loss observed below 100 °C to the evaporation of free fluids, while the weight loss between 100 °C and 200 °C is primarily associated with the release of bound fluids from the polymeric chains [35]. Additionally, the weight loss observed below 100 °C suggests that the mucoadhesive film retains thermal stability at temperatures below this threshold, supporting its suitability for application in the treatment of cervicovaginal lesions [36,37].

### 2.4. FTIR Analysis

**Figure 5:**
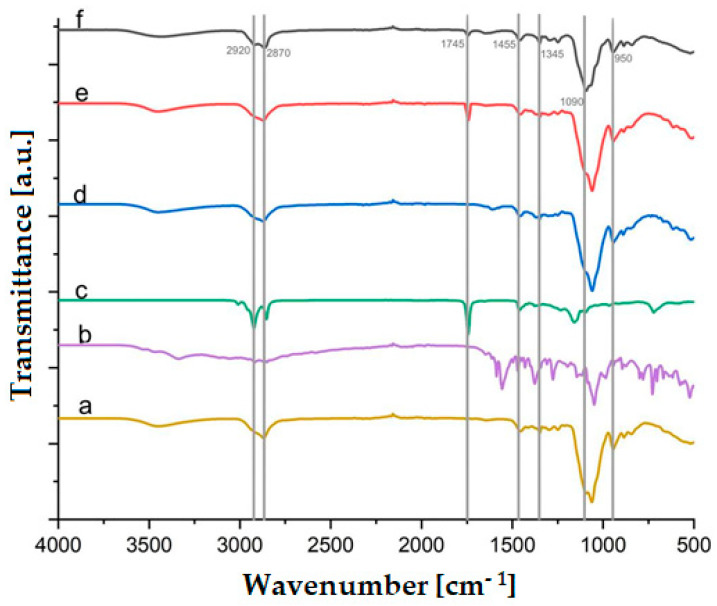
FTIR spectrum of (a) film C (21 wt. % of HPMC and 79 wt. % of PEG 400), (b) ketorolac, (c) retinyl palmitate, (d) film G (20.75 wt. % of HPMC, 77.16 wt. % of PEG 400 and 2.06 wt. % of ketorolac), (e) film E (20.90 wt. % of HPMC, 78.27 wt. % of PEG 400, and 0.83 wt. % of retinyl palmitate), and (f) film H (20.34 wt. % of HPMC, 76.80 wt. % of PEG 400, 0.82 wt. % of retinyl palmitate, and 2.04 wt. % of ketorolac).

The FTIR spectra of retinyl palmitate, ketorolac, control polymeric films, ketorolac-loaded films, and films with or without retinyl palmitate are displayed in Figure 5. The spectra reveal the H-C-H stretching at 2920 cm^−1^, accompanied by asymmetrical bending at 1455 cm^−1^. The C-H stretching is observed at 2870 cm^−1^, while the peak at 1745 cm^−1^ indicates the C=O group from the retinyl palmitate molecule (films E and F). The peak at 1375 cm^−1^ corresponds to the C-N group from ketorolac, and the C-O-C group exhibits stretching and bending movements characteristic of the original film (film C). Notably, the C-O-C stretching appears at 1050 cm^−1^ in samples a, d, and e but shifts to 1090 cm^−1^ in sample f, which can be attributed to intermolecular forces such as hydrogen bonding. Additionally, spectra a, d, e, and f exhibit an absorption band at 1345 cm^−1^, which is associated with the C-H bending vibrations.

### 2.5. Swelling and Weight Loss from Films

**Figure 6:**
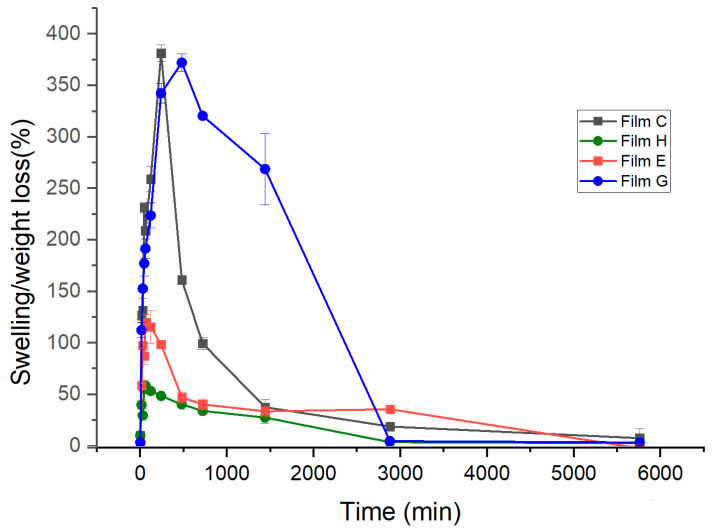
Changes in Swelling and Weight Loss of Polymeric Film C (21 wt. % of HPMC and 79 wt. % of PEG 400), H (20.34 wt. % of HPMC, 76.80 wt. % of PEG 400, 0.82 wt. % of retinyl palmitate, and 2.04 wt. % of ketorolac), E (20.90 wt. % of HPMC, 78.27 wt. % of PEG 400, and 0.83 wt. % of retinyl palmitate) and G (20.75 wt. % of HPMC, 77.16 wt. % of PEG 400, 77.16 wt. % of retinyl palmitate, and 2.06 wt. % ketorolac).

Figure 6 presents the results of weight loss and swelling tests for films C, E, G, and H, providing valuable insights into the material’s behavior in biological environments. Based on the statistical analysis conducted, significant differences were observed in the swelling and weight loss of the polymeric films. These results indicate variations in absorption capacity among the different samples analyzed.

Notably, the swelling occurs in film C, increasing by more than 400 times its original weight, indicating an exceptional moisture uptake capacity. This initial swelling phase is significantly more effective than the weight loss phase, suggesting that the material reaches a hydration threshold quickly before stabilizing.

The weight loss process extends over three days, indicating the material’s gradual degradation. This degradation facilitates the release of active substances into the surrounding environment and promotes favorable interactions with cells, potentially enhancing cellular uptake. Film G exhibits behavior similar to film C but shows approximately 20% less absorption due to its partially occupied capacity during ketorolac absorption. In the case of films containing retinol, specifically film E and film H, the hydrophobic characteristics of retinol hinder water uptake. This results in swelling levels of approximately 100% for film E and 70% for film H, which shows lower swellability due to the presence of ketorolac.

Our findings are partially consistent with previous studies that reported similar swelling behaviors in polymeric systems; however, the degree of swelling and the duration of weight loss may differ based on factors such as polymer composition, cross-linking density, and environmental conditions [27].

### 2.6. In Vitro Release Test

**Figure 7:**
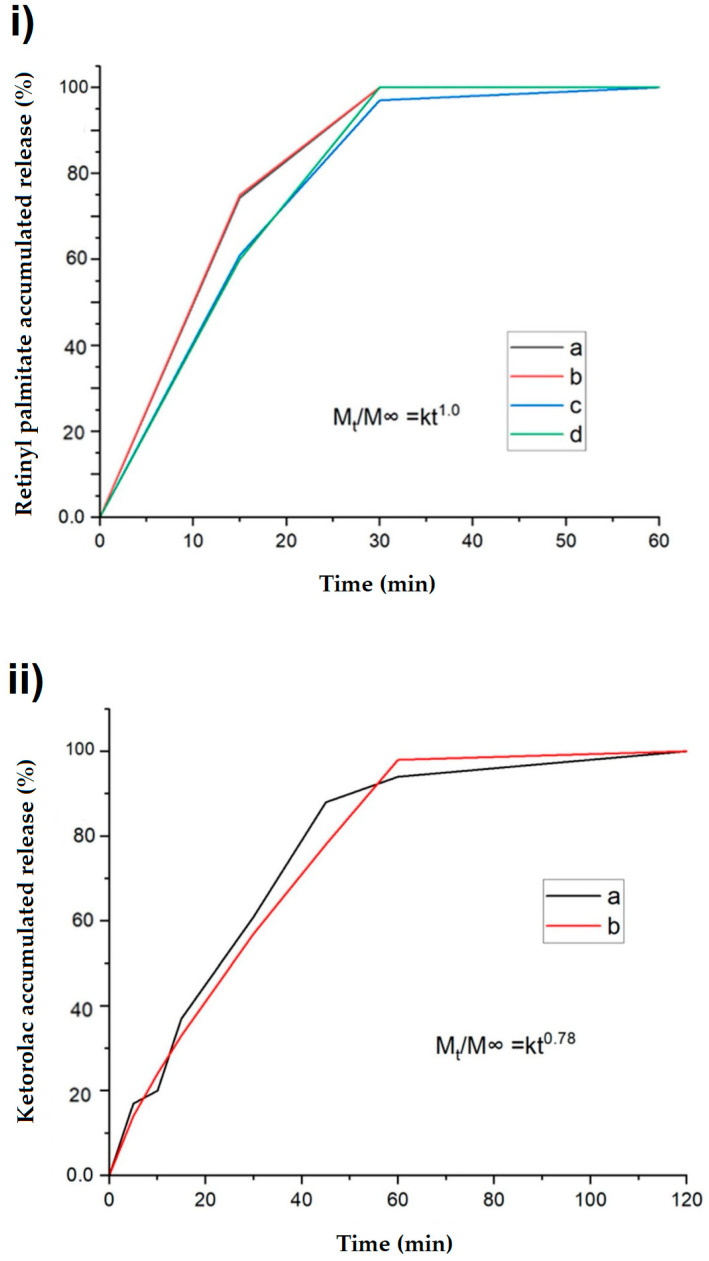
(**i**) Accumulated release percentage of films with HPMC and PEG 400: (a) film B measured (14.97 wt. % of HPMC, 84.30 wt. % of PEG 400, and 0.73 wt. % of retinyl palmitate); (b) film B calculated; (c) film D measured (20.94 wt. % HPMC, 78.38 wt. % PEG 400, and 0.67 wt. % retinyl palmitate); (d) film D calculated. (**ii**) Accumulated release percentage of HPMC and PEG 400 polymeric films loaded with ketorolac: film G (20.75 wt. % of HPMC, 77.16 wt. % of PEG 400, 77.16 wt. % of retinyl palmitate, and 2.06 wt. % ketorolac): (a) empirical and (b) calculated release.

There is a correlation between the drug release process and HPMC concentration, as observed using the UV-VIS test: reducing the amount of HPMC results in faster drug release. Our current data are supported by previous research [38,39]. Increasing the amount of HPMC enhances the system’s swelling, prolonging the dissolving and release period, as shown in Figure 7i. The release of ketorolac was characterized by comparing whether the hydrophilic nature of the molecule and changes in the characteristics of the medium influence the release of the HPMC and PEG 400 system. In this case, a greater retention of the active substance in the system can be observed (Figure 7ii), which is attributable to the system’s affinity for water and related molecules. Compared to the previous release, the total release was achieved 120 min later [38]. The regression coefficients for films B, D, and G were 0.9999, 0.9988, and 0.9802, respectively, demonstrating the reliability of the theoretical model based on the power law for polymeric thin films.

The theoretical model was based on the power law for polymeric thin films, resulting in an exponent *n* ranging from 0.5 to 1.0, depending on the release rate [39]. To match the empirical release rates, *n* = 0.78 was used for ketorolac and *n* = 1 for retinol, indicating that both exhibit anomalous transport behavior. In particular, a value of *n* = 1 indicates controlled drug release via polymer swelling, while *n* = 0.5 suggests a diffusion-controlled release. Values of *n* between 0.5 and 1.0 suggest the presence of both phenomena, which is interpreted as anomalous transport behavior [39].

### 2.7. Contact Angle Test

**Figure 8:**
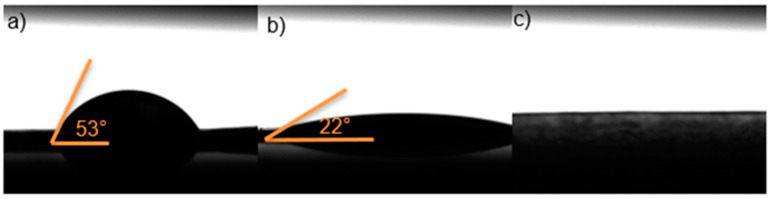
Contact angle of films: (**a**) film A (15 wt. % of HPMC and 85 wt. % of PEG 400), (**b**) film D (20.94 wt. % of HPMC, 78.38 wt. % of PEG 400, and 0.67 wt. % of retinyl palmitate), and (**c**) film I (26 wt. % of HPMC and 74 wt. % of PEG 400).

According to the results obtained with the contact angle study (Figure 8) of the three films, increasing the amount of HPMC results in a smaller contact angle. All three samples showed angles smaller than 90°, indicating the system is hydrophilic. In these tissues (cells), substrate interaction could suggest biocompatibility. In Figure 8c, the contact angle could not be measured because it is hyper-hydrophilic, which could cause an imbalance in drug release. This finding agrees with a previous report indicating that a viable system should have a contact angle between 0 and 90° [40].

### 2.8. Film Adhesion to Tissue and Glide Test

**Figure 9:**
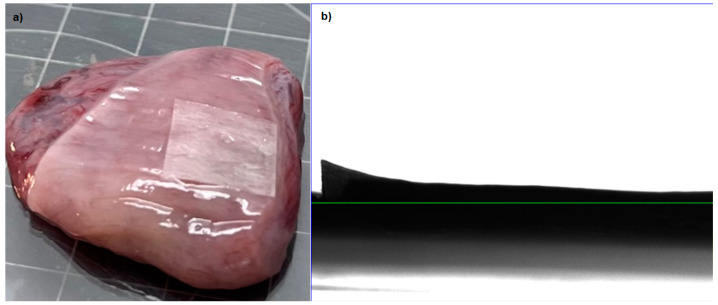
Adhesion of polymeric film to the uterus tissue: (**a**) the tissue used during testing, (**b**) Image of the film with the tissue, captured using a Drop Shape Analyzer.

Figure 9 shows the interaction between the polymeric film and the tissue, confirming the previous results. Since no separation is present, the film is completely attached; a slight difference can be seen in the corner due to the swelling after applying the vaginal fluid simulant. A transformation occurs following the interaction between the tissue, the polymeric film, and the vaginal fluid simulant; according to Teworte et al., with longer interaction, the system loses its original form and becomes a hydrogel [41].

**Figure 10:**
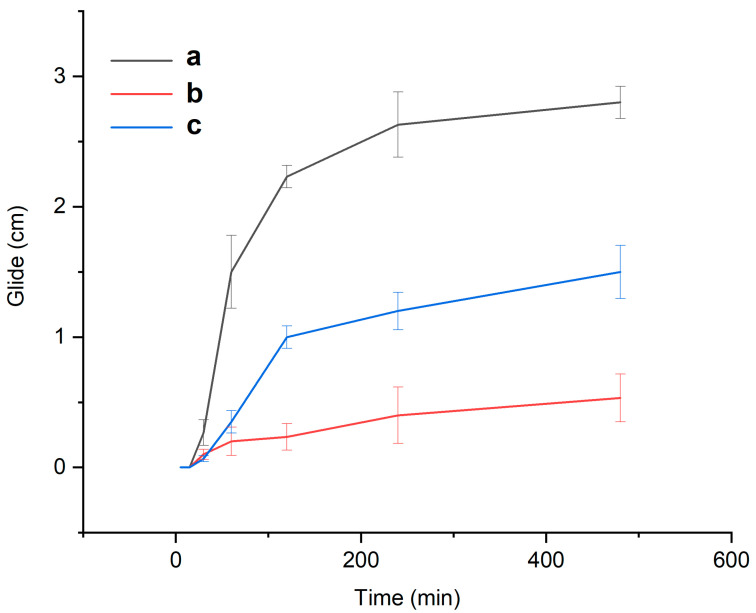
Glide test: (a) film C (21 wt. % of HPMC and 79 wt. % of PEG 400); (b) film E (20.90 wt. % of HPMC, 78.27 wt. % of PEG 400, and 0.83 wt. % of retinyl palmitate); (c) film H (20.34 wt. % of HPMC, 76.80 wt. % of PEG 400, 0.82 wt. % of retinyl palmitate, and 2.04 wt. % of ketorolac).

Figure 10 shows the glide of the polymeric films loaded with active molecules or on their own. The film E (containing retinyl palmitate) exhibited less glide compared to the others, which can be attributed to the lumpy topography observed in the FESEM characterization (see Figure 4). In the case of the ketorolac-loaded films, they glided only half as far as the unloaded film. Although the film by itself has many pores, which, according to Gyarmati, may help achieve a specific compressive force, this force transforms into interfacial interactions when in contact with the mucus layer, resulting in deformation [42] as it loses its original form and becomes a mucoadhesive gel. As observed, the lumpy topography helps maintain better adhesion to the mucus barrier than the porous structure. Overall, all three films slid less than 4 cm, which is less than half the average vaginal canal length of 9 cm [43].

### 2.9. Cellular Viability

**Figure 11:**
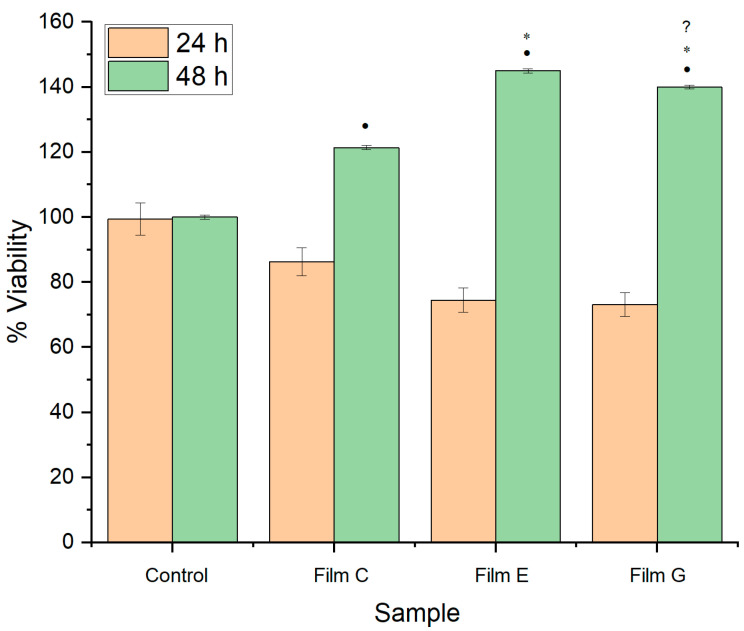
MTT assay of 24 h and 48 h for films C (21 wt. % of HPMC and 79 wt. % of PEG 400), E (20.90 wt. % of HPMC, 78.27 wt. % of PEG 400, and 0.83 wt. % of retinyl palmitate), and G (20.75 wt. % of HPMC, 77.16 wt. % of PEG 400, and 2.06 wt. % of ketorolac). •: significant difference compared to control, ∗: significant difference compared to film, ?: significant difference compared to film with retinyl palmitate. (ρ = 0.05, comparison is made between control and samples of the same day).

Cellular viability was evaluated for films containing retinyl palmitate or ketorolac at 24 and 48 h. Figure 11 shows that at 24 h, cell viability is minimally affected, but recovery is observed at 48 h. The films containing retinyl palmitate and ketorolac exhibited the highest percentage of viability at 48 h.

Cervical cancer (CC) poses a significant public health challenge globally, including in Mexico [1]. Persistent Human Papillomavirus (HPV) infection is widely recognized as the primary risk factor for the development of CC [2]. Cervicovaginal lesions frequently occur in association with sexual activity. Moreover, it is well-documented that microabrasions in the cervical epithelium during sexual activity facilitate HPV infections.

Previous reports have highlighted the importance of Cellular Retinol Binding Protein 1 (CRBP1) protein expression and retinoids as crucial molecular mechanisms involved in the basal cell layer of healthy cervical epithelium. However, a lack of CRBP1 expression and low retinol serum levels are more commonly observed in CC [44]. Recent findings indicate that administering retinol in conjunction with cisplatin can effectively eliminate a high percentage of cancerous cells [45]. These findings suggest that utilizing the HPMC-PEG-retinyl palmitate system could be a promising and innovative approach for treating cervicovaginal lesions.

## 3. Materials and Methods

### 3.1. Materials

In the present work, various materials were used, including HPMC (viscosity 2600–5600 cP, Sigma-Aldrich, Ciudad de México, México), dichloromethane (Sigma-Aldrich, Ciudad de México, México), methanol (Sigma-Aldrich, Ciudad de México, México), albumin from fetal serum (Sigma-Aldrich, Ciudad de México, México), lactic acid (Sigma-Aldrich, Ciudad de México, México), urea (Sigma-Aldrich, Ciudad de México, México), PEG 400, glycerol (Alfa Aesar, Monterrey, Nuevo León, México); retinyl palmitate (Spring Valley, Monterrey, Nuevo León, México), ketorolac (LIOMONT, Ciudad de México, México); sodium chlorate, acetic acid, hydrochloric acid (J.T. Baker, Monterrey, Nuevo León, México); potassium hydroxide (MCB, Monterrey, Nuevo León, México); calcium hydroxide (Fermont); and glucose (Fagalab, Monterrey, Nuevo León, México).

### 3.2. Fabrication of Polymeric Films

The fabrication of polymeric films followed the method previously reported by Grameen et al. [9]. Briefly, a mixture of HPMC, PEG 400, and dichloromethane in methanol at a ratio of 3:1 was left uncovered under magnetic stirring at 5000 rpm for 1 h using the solvent evaporation technique (see Table 1). Dichloromethane was added, followed by the slow incorporation of HPMC while stirring with a magnetic stirrer. PEG 400 and retinyl palmitate were added sequentially, with stirring between each addition. Then, methanol was added, and the mixture was stirred until uniform. The system was dried in a Pyrex petri dish (area of 21 cm^2^) for 48 h at 37 °C [9]. For the loading of ketorolac, dissolved in 1 mL of ethanolic solution, the previously obtained HPMC and PEG400 film was cut into portions, and ketorolac was added to each portion using absorption and complexation methods. The samples were sterilized by exposing them to UV light for 15 min on each side.

### 3.3. Films’ Characterization

The vaginal fluid simulant was prepared according to Owen’s methodology [46], consisting of distilled water (900 mL), sodium chloride (3.5 g), potassium hydroxide (1.4 g), calcium hydroxide (0.22 g), albumin (0.018 g), lactic acid (2 mL), acetic acid (1 mL), glycerol (0.16 mL), urea (0.4 g), glucose (5 g), and hydrochloric acid to adjust the pH to 4.5.

Morphological analysis and film thickness measurements were carried out using Scanning Electron Microscopy (SEM). The SEM characterization was performed with a FESEM Hitachi SU5000, equipped with a base of 51 mm and operated at a working distance of 12 mm. The analysis was conducted in low-pressure mode (30 Pa) with an accelerating voltage of 15 kV, enabling both backscattered and secondary electrons.

Thermogravimetric analysis (TGA) was conducted to monitor the film’s dehydration using a TA Instruments SDT Q600 at temperatures ranging from 20 °C to 200 °C.

Infrared (IR) spectra were obtained using a Nicolet 6700 instrument to determine the functional groups in the film. The films were cut and placed on an ATR adapter with a germanium crystal for analysis. All spectra were registered over 100 scans with a resolution of 16 cm^−1^, and all samples were scanned in the range of 550–4000 cm^−1^.

For the swelling test, 1 × 1 cm^2^ sections were cut in triplicate from the film. The initial and final weights were measured. The samples were immersed in a PBS solution for 0, 5, 15, 30, 60, 120, 360, and 480 min. The following equation was used to obtain the swelling percentage (Ps):

(1) $$\mathrm{Ps}=\frac{\mathrm{Ws}-\mathrm{Wd}}{\mathrm{Wd}}\times 100$$

where Ps is the swelling percentage, Wd the initial weight, and Ws the final weight.

### 3.4. Release Test

The wavelength used for the retinyl palmitate release test was measured using UV-Vis spectroscopy. The UV-Vis spectra were obtained with a Thermo Scientific NanoDrop 2000 Spectrophotometer, covering a range from 190 to 840 nm, with a focused wavelength of 325 nm [47]. A calibration curve was constructed using increments of 5 mM, ranging from 0 to 15 mM, by dissolving retinyl palmitate in ethanol.

The calibration curve for ketorolac was constructed using wavelengths of 222, 224, and 272 nm, with concentrations ranging from 0 to 120 mM in 20 mM increments, prepared by dissolving ketorolac in distilled water. The release of ketorolac from the film was then measured at a wavelength of 222 nm.

Small portions of the film were cut, approximately 1 cm^2^, and exposed to a vaginal fluid simulant solution with pH 4.5 at 37 °C. Table 2 shows the linearity equation, release rate, correlation coefficient, limit of detection (LOD), and limit of quantification (LOQ) for the release test.

The theoretical calculation of retinyl palmitate release followed the power law equation (Korsmeyer–Peppas model) expressed as:

Mt/M∞ =k t^n (2)

where M∞ represents the total amount of drug loaded into the system, Mt is the amount of the active molecule released at time t, k is a constant dependent on the system’s variables (also known as the release rate constant), and n is a parameter that varies according to the characteristics and shape of the release matrices. For polymeric thin films, n typically ranges from 0.5 to 1.0 [39].

### 3.5. MTT Assay

The MTT assay (3-[4,5-dimethylthiazol-2-yl]-2,5 diphenyl tetrazolium bromide) was used to evaluate cellular metabolic activity. This colorimetric analysis measures enzyme activity, resulting in a variation of the purple color of the MTT dye.

Fibroblasts were obtained from a primary culture and used to evaluate the cytotoxicity of the HPMC and PEG 400 polymeric film (permission from the research ethics committee CEI-2023-2-148). The fibroblasts were cultured in DMEM media and distributed at 5000 cells/well. They were then placed in a humidified incubator with 5% CO_2_ at 37 °C. The films were sterilized using UV radiation. The cells were deposited in wells containing the film, film with retinyl palmitate, and film with ketorolac and subsequently incubated for 24 h and 48 h. The MTT solution (5 mg/mL in PBS) was added to the wells and incubated for 4 h at 37 °C. After the MTT was removed, 100 µL of DMSO was added to dissolve the formazan crystals formed. Cell viability (%) was measured at λ = 570 nm [48,49].

### 3.6. Glide Test

**Figure 12:**
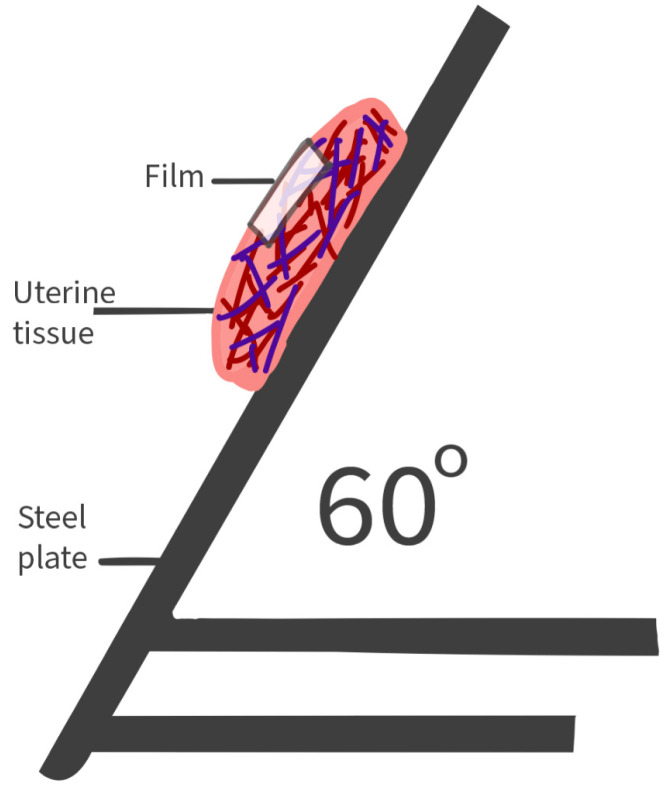
Sliding system for measuring film H adhesion in porcine uterine tissue.

The contact angle and adhesion images were taken with a Krüss system DSA30. The system’s adhesion was first evaluated through the contact angle measurement and by assessing glide using a system, as shown in Figure 12. Porcine uterine tissue was obtained post-slaughter, cut into 5 cm pieces, and stored for 6 h following the Teworte methodology [41].

### 3.7. Statistical Analysis

The data obtained were evaluated for statistical significance using one-way ANOVA (Tukey’s Post Hoc Test with 95% confidence interval).

## 4. Conclusions

The characteristics such as size, shape, and distribution of porosity of the current films, can be altered based on HPMC concentrations. The humidity and contact angle may also vary depending on the amount of polymer used. Interestingly, upon contact with fluids, the film transforms into a mucoadhesive gel, swelling for at least 100 min and degrading within approximately 3–4 days. Intermolecular hydrogen bonds form when the polymeric film is loaded with different active molecules, with the active molecules occupying the pores. This results in a lumpier film that adheres better to the mucus barrier. Furthermore, the present film demonstrates biocompatibility, whether with or without retinyl palmitate. Drug delivery using this polymeric system is supported by the Higuchi equation.

In summary, this study represents emerging proof-of-concept research utilizing an HPMC-retinyl palmitate film system to treat cervicovaginal lesions. It is plausible to hypothesize that this system could potentially be extended to treat skin lesions as well.

## Figures and Tables

**Table 1:** Composition of polymeric films.

Film	HPMC (wt. %)	PEG 400 (wt. %)	Dichloromethane (mL)	Methanol (mL)	Retinyl Palmitate (wt. %)	Ketorolac (wt. %)
**A**	15.00	85.00	15	5	0	0
**B**	14.97	84.30	15	5	0.73	0
**C**	21.00	79.00	15	5	0	0
**D**	20.94	78.38	15	5	0.67	0
**E**	20.90	78.27	15	5	0.83	0
**F**	20.88	78.10	15	5	1.00	0
**G**	20.75	77.16	15	5	0	2.06
**H**	20.34	76.80	15	5	0.82	2.04
**I**	26.00	74.00	15	5	0	0

**Table 2:** Linearity equation, release rate, correlation coefficient, limit of detection (LOD), and limit of quantification (LOQ) of release test.

Drug	Linearity Equation	Release Rate (μg/mL/min)	Correlation Coefficient	LOD (μg/mL)	LOQ (μg/mL
Retinyl palmitate	y = 0.0033x + 0.2882	516 ± 0.23	0.9559	237.18	696.96
Ketorolac	y = 0.0088x − 0.55	433.33 ± 0.137	0.9682	51.37	155.68
